# Open Double Mallet Lesion of the Ring Finger with Concomitant Little Finger Fracture: A Case Report

**DOI:** 10.3390/diagnostics16091248

**Published:** 2026-04-22

**Authors:** Suguru Yokoo, Takahiro Toriyama, Yukimasa Okada, Chuji Terada

**Affiliations:** 1 Department of Orthopaedic Surgery, Fukuyama City Hospital, Hiroshima 721-8511, Japan; 2 Department of Orthopaedic Surgery, Chikamori Hospital, Kochi 780-8522, Japan

**Keywords:** mallet finger, open double mallet lesion, bony mallet, pull-out fixation

## Abstract

**Background and Clinical Significance**: Mallet finger is a common injury of the extensor mechanism at the distal interphalangeal (DIP) joint; however, open double mallet lesions are rare and may present a complex reconstruction challenge. **Case Presentation**: A 15-year-old male high school student who sustained an open injury to the left ring and little fingers after a high-energy buggy accident. The ring finger showed an open double mallet lesion in which the extensor tendon remained attached to a tiny avulsion fragment, and a separate dorsal base fragment was also present. The adjacent little finger had a concomitant open fracture with substantial soft tissue injury. Emergency surgery was performed on the day of the injury. For the ring finger, reduction of the tendon-attached avulsion fragment and separate dorsal base fragment was achieved using extension-block pinning, transarticular DIP pinning, and pull-out fixation over a volar button. For the little finger, cross-pinning was performed because the distal fragment was too small for stable non-transarticular fixation. Serial radiographs showed maintained alignment and progressive healing. At the final follow-up, 21 months after the injury, residual deformity and limitation of DIP motion remained; however, no infection, major skin complications, or nail deformity were observed. The little finger DIP joint became ankylosed, whereas some residual mobility remained in the ring finger DIP joint. Despite persistent functional limitations, the patient was able to continue school attendance and percussion-related activities. **Conclusions**: This case highlights that in an open double mallet lesion, disruption of both the tendon-attached fragment and its bony bed should be considered, and stabilization of the base may be useful in selected injury patterns before definitive tendon-side repair.

## 1. Introduction

Mallet finger is a common injury of the extensor mechanism at the distal interphalangeal (DIP) joint and is generally classified as either tendinous or bony, depending on the presence of an avulsion fracture at the dorsal base of the distal phalanx [1,2]. In most cases, mallet injuries are closed and present a relatively simple injury pattern [3,4]. In routine bony mallet injuries, treatment strategies are usually guided by fragment size, DIP joint subluxation, and degree of displacement [5].

**Figure 1:**
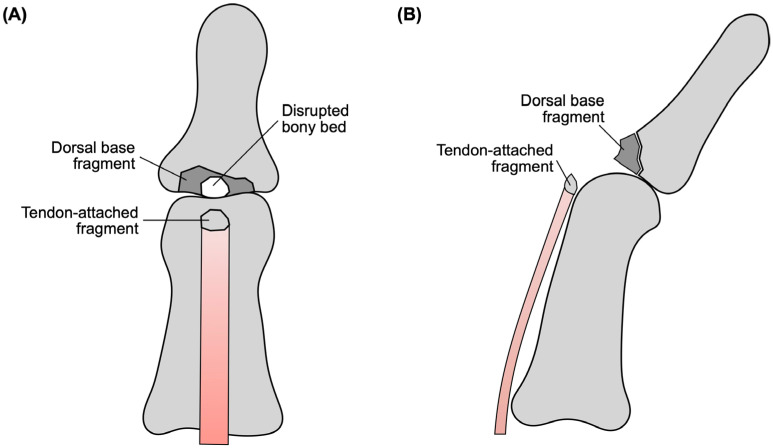
Schematic illustration of a double-level mallet lesion. (**A**) Frontal view showing the tendon-attached fragment, disrupted bony bed, and dorsal base fragment. (**B**) Lateral view showing the separate dorsal base and tendon-attached fragments.

A more unusual pattern is the so-called double mallet lesion, in which a tendon-attached tiny avulsion fragment coexists with a separate dorsal base fragment [6,7] (Figure 1). As the lesion involves both the extensor tendon insertion and the underlying osseous bed, it is more complex than an ordinary single-fragment bony mallet injury. A preoperative diagnosis may also be difficult, particularly when the tiny tendon-attached fragment is not clearly visible on plain radiographs [6,7].

Open mallet-type injuries are uncommon and may be associated with substantial soft tissue injury and more complex disruption of the extensor mechanism [8,9,10]. In such situations, preoperative plain radiographs alone may not fully demonstrate the actual relationship between the extensor tendon and osseous fragments. Therefore, computed tomography (CT) and careful intraoperative assessment may be important for clarifying fragment configuration and planning an appropriate repair strategy [11,12].

In the present case, the ring finger showed an open double mallet lesion in which the extensor tendon remained attached to a tiny avulsion fragment, and a separate dorsal base fragment was also present. In addition, the adjacent little finger sustained a concomitant open fracture with substantial soft tissue injury. This combination created a challenging reconstruction situation in which both anatomical reduction and restoration of extensor continuity had to be considered. The specific novelty of this case lies not only in the open double-level injury pattern itself, but also in the recognition that both the tendon-attached fragment and the bony bed were disrupted, which influenced the reconstructive strategy. To our knowledge, reports of open double mallet lesions are extremely limited, and practical guidance for this injury configuration remains sparse. We report this case to highlight the diagnostic and surgical implications of this injury configuration, with particular emphasis on the role of imaging, intraoperative fragment identification, and staged fixation in the management of an open double mallet lesion with concomitant little finger trauma.

## 2. Case Presentation

### 2.1. Ethics and Setting

This study adhered to the Declaration of Helsinki and was approved by the Institutional Review Board of Fukuyama City Hospital (approval no. 846; 29 November 2024). Written informed consent has been obtained from the patient’s guardian to publish this paper. This is a single-patient case report from a tertiary referral hospital.

### 2.2. Patient Information and Injury Mechanism

A 15-year-old male high school student was brought to our hospital on the day of the injury. He was functionally ambidextrous, using his right hand for writing and using chopsticks, and his left hand for sports activities and playing percussion in a school music club. He had no relevant medical history. While riding in a buggy with his father at an amusement park, the vehicle became immobile on a slope. It then unintentionally restarted, rolled down the slope for approximately 15 m, crossed a fence, and fell by approximately 5 m. During this accident, he sustained an open injury to the left ring and little fingers.

### 2.3. Initial Examination and Imaging Findings

**Figure 2:**
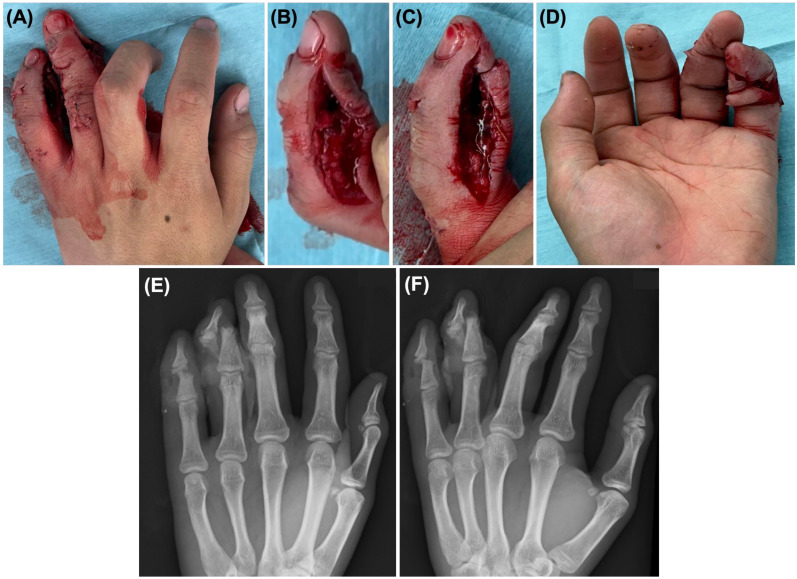
Preoperative photographs and radiographs of the left hand. (**A**) Dorsal view showing open injuries involving the ring and little fingers. (**B**) Radial-side view emphasizing ring finger injury. (**C**) Radial-side view emphasizing little finger injury. (**D**) Palmar view. (**E**) Posteroanterior radiograph and (**F**) oblique radiograph showing associated phalangeal fractures.

On initial examination, both the ring and little fingers showed open soft tissue injuries around the DIP region without apparent vascular compromise. The ring finger had an approximately 5 cm radial laceration. The little finger had an approximately 5 cm radial laceration and an additional approximately 4 cm volar laceration continuous with the ulnar side, indicating more extensive soft tissue disruption (Figure 2). No obvious complete tendon rupture was clinically identified. Initial sensory examination was limited by pain, the acute trauma setting, and the presence of multiple associated injuries, and no obvious complete sensory loss was recognized. In addition to hand injuries, the patient had multiple associated injuries, including splenic injury with intraperitoneal bleeding, rib fractures, pulmonary contusion, pneumothorax, facial trauma, and concussion, all of which were managed conservatively.

**Figure 3:**
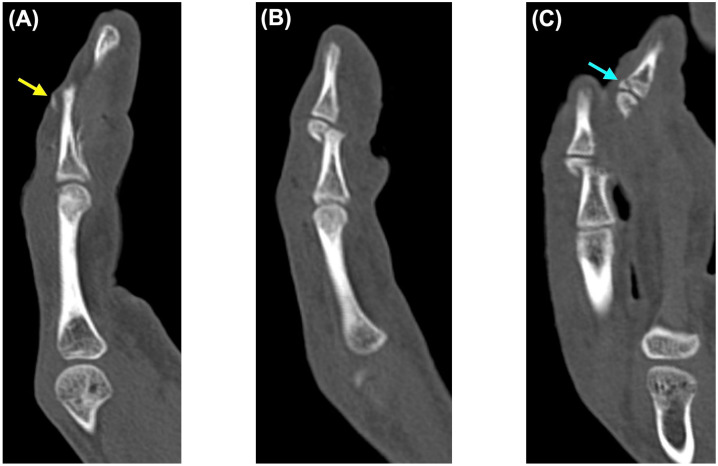
Preoperative computed tomography of the left ring and little fingers. (**A**) Sagittal image of the ring finger showing a tiny dorsal avulsion fragment at the base of the distal phalanx, consistent with bony mallet injury (yellow arrow). (**B**) Sagittal image of the little finger showing a middle phalanx fracture with a small distal fragment. (**C**) Coronal reconstructed image showing the spatial relationship between the ring and little finger injuries. The blue arrow indicates the bone fragment at the base of the distal phalanx of the ring finger.

Plain radiographs and CT of the left hand were obtained (Figure 2 and Figure 3). Imaging revealed a bony mallet-type injury of the ring finger, characterized by a tiny dorsal avulsion fragment at the base of the distal phalanx. CT also demonstrated a separate dorsal base fragment of the distal phalanx, raising suspicion of a double-level injury pattern preoperatively. In the little finger, imaging demonstrated a middle phalanx fracture with a small distal fragment. Although CT clarified the osseous fragment configuration and the spatial relationship between the ring and little finger injuries, definitive understanding of the tendon attachment and disruption of the bony bed was achieved intraoperatively.

### 2.4. Operative Findings and Procedure

Emergency surgery was performed on the day of the injury under upper extremity conduction anesthesia, with the patient in the supine position under fluoroscopic guidance. Irrigation and debridement were performed first. Contamination of both wounds was mild, and both fingers were thoroughly irrigated with saline. No arterial injury was identified in either finger, and no definite nerve transection was confirmed.

**Figure 4:**
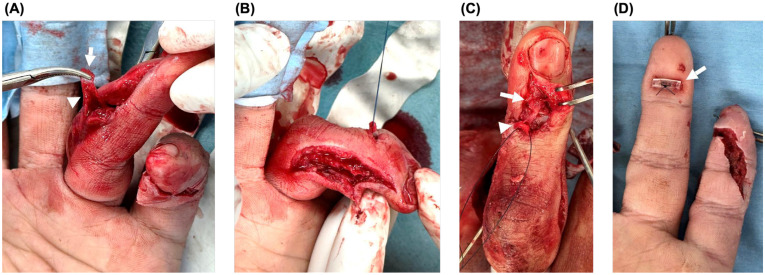
Intraoperative findings and tendon-side reconstruction of the ring finger. (**A**) The tendon-attached avulsion fragment was identified through the radial wound (arrow), while the extensor tendon remained attached to the tiny fragment (arrowhead). (**B**) The tendon-attached fragment was transposed toward the dorsal wound for reduction and repair. (**C**) Intraoperative view showing both the tendon-attached tiny fragment (arrowhead) and the separate dorsal base fragment (arrow). (**D**) Pull-out fixation was completed, with the suture tied over a button on the volar side (arrow).

For the ring finger, the fracture configuration was assessed through the open wounds. The extensor tendon remained attached to a tiny avulsion fragment identified through the radial wound. This tendon-attached fragment was then brought out through the dorsal wound, where a separate dorsal base fragment of the distal phalanx was also identified. Thus, not only the tendon-attached avulsion fragment, but also the bony base to which it should normally be reduced, was disrupted (Figure 4).

Because the fracture configuration was highly unstable, a 1.1-mm Kirschner wire (K-wire) was first inserted from the ulnar side of the base of the middle phalanx to obtain provisional stability and fracture fixation. After clarifying the fragment configuration, a 4-0 nylon suture was placed in the tiny tendon-attached avulsion fragment.

**Figure 5:**
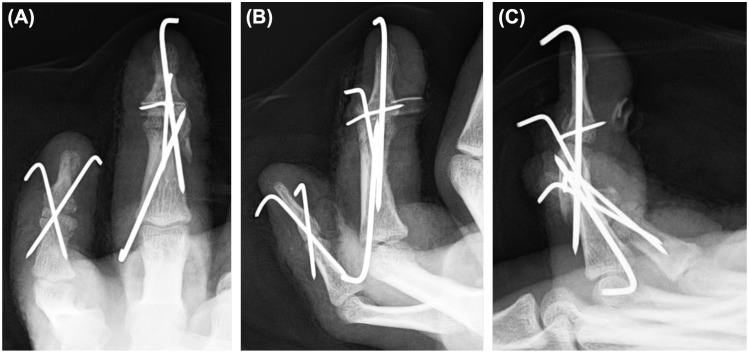
Immediate postoperative radiographs of the left ring and little fingers. The ring finger was stabilized using transarticular DIP joint pinning, extension-block pinning, additional K-wire fixation of the dorsal base fragment, and pull-out fixation of the tendon-attached fragment. The little finger was stabilized with cross K-wire pinning. (**A**) Posteroanterior view. (**B**) Oblique view. (**C**) Lateral view.

A 0.7-mm K-wire was then used to align the tendon-attached avulsion fragment, separate dorsal base fragment, and main distal phalanx fragment. An extension-block pin was inserted dorsally with the DIP joint in flexion using a 0.9-mm K-wire. Transarticular DIP joint pinning was subsequently performed with a 1.1-mm K-wire inserted from the distal phalanx. A bone tunnel was then created in the distal phalanx with a 1.1-mm K-wire, and the previously placed 4-0 nylon suture was pulled out to the volar side and tied over a Nelaton tube as a button, thereby completing the pull-out fixation (Figure 4 and Figure 5).

For the little finger, the distal fragment was too small for stable fixation that would avoid crossing the DIP joint. A non-transarticular fixation method was considered preferable; however, this was impractical because of the fragment size. Therefore, cross-pinning through the distal phalanx was performed from the distal side using two 0.9-mm K-wires. Given the small distal fragment and the local soft tissue injury, distal cross-pinning was selected as a pragmatic strategy to provide stability while protecting the soft tissue.

**Figure 6:**
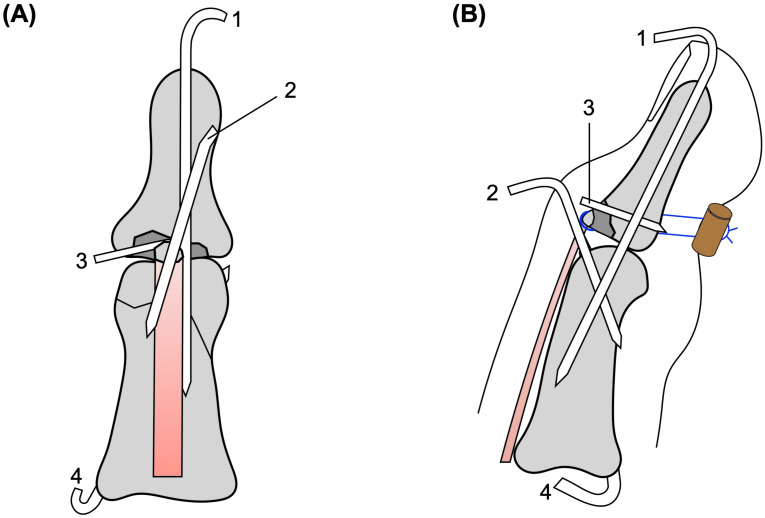
Schematic illustration of the fixation construct. (**A**) Frontal view showing the K-wire configuration of the ring finger. (**B**) Lateral view showing transarticular DIP joint pinning, extension-block pinning, K-wire fixation of the dorsal base fragment, middle phalanx provisional stabilization and fixation, and pull-out fixation of the tendon-attached fragment over a volar button. 1, transarticular DIP joint pin; 2, extension-block pin; 3, K-wire for dorsal base fragment fixation; 4, K-wire for middle phalanx provisional stabilization and fixation.

After the fixation of both fingers, the wounds were irrigated again, closed, and dressed. Postoperative radiographs demonstrated acceptable alignment and pin configuration (Figure 5). A schematic illustration of the fixation construct is shown in Figure 6.

### 2.5. Postoperative Management

Occupational therapy was initiated on postoperative day 3. For wound protection, a volar splint was applied for 1 week after surgery; however, it was removed during rehabilitation sessions to allow range-of-motion exercises. Proximal interphalangeal (PIP) joint range-of-motion exercises, including passive motion, were started immediately after the surgery, whereas the DIP joints were protected during the period of K-wire fixation. Heavy gripping or forceful use was not allowed during this period. The postoperative course is summarized in Table 1.

At an early postoperative follow-up approximately 2 weeks after surgery, pain was mild, with a numerical rating scale score of 0 at rest and 2 during motion. The Disabilities of the Arm, Shoulder and Hand (DASH) disability/symptom score was 39 at that time, with music and work module scores of 44 and 31, respectively.

**Figure 7:**
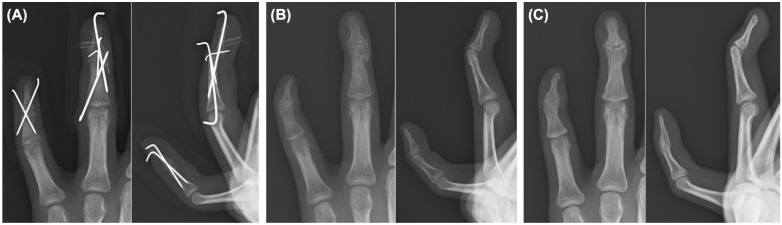
Serial radiographs of the left ring and little fingers. (**A**) Radiographs obtained at 6 weeks, before hardware removal, showing maintained alignment. (**B**) Radiograph obtained at 3 months, showing progression of fracture healing. (**C**) Final radiograph obtained at approximately 21 months, showing union and final alignment of the ring and little fingers.

Six weeks after surgery, all K-wires and the pull-out suture were removed. Serial radiographs demonstrated maintained alignment. After hardware removal, both active and passive motion were generally allowed, although excessive loading and heavy gripping were still discouraged. Approximately 3 months after surgery, fracture healing progressed, and daily activities were largely recovered, although residual DIP joint stiffness remained. The patient returned to school soon after discharge and resumed music-related activities three months after surgery. Grip strength was 37.5 kg on the unaffected side and 25.7 kg on the affected side at this stage. At 9 months, residual functional impairment persisted, although grip strength improved to 38.4 kg on the unaffected side and 20.9 kg on the affected side (Figure 7; Table 1).

### 2.6. Final Outcome

At the final follow-up, approximately 21 months after the injury, residual deformity and limitation of DIP joint motion remained. The ring finger showed a DIP extension lag of 40° and flexion of 45°, whereas the little finger showed a DIP extension lag of 10° and flexion of 20°. In contrast, PIP joint motion was preserved in both digits, and a full composite grip was possible. Grip strength at the final follow-up was 41.3 kg on the unaffected side and 30.6 kg on the affected side. Both fingers retained DIP flexion strength, whereas the extension strength was weaker, particularly in the little finger. Coronal deformity was present, with approximately 20° valgus deformity of the ring finger DIP joint and 30° varus deformity of the little finger, accompanied by an occasional crossover tendency of the little finger, which was self-controlled by blocking with the ring finger. Residual sensory dullness persisted over the dorsoulnar aspect of both digits. No infections, nail deformities, or major skin complications were observed.

**Figure 8:**
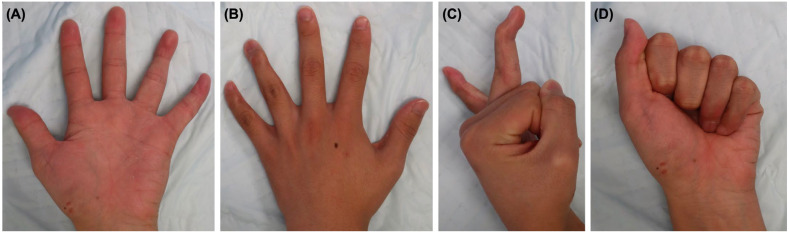
Clinical appearance and range of motion of the left hand at the final follow-up (21 months). (**A**) Volar view. (**B**) Dorsal view. (**C**) Lateral view showing the resting posture and residual deformity of the ring and little fingers. (**D**) Active flexion at the final follow-up.

The patient reported difficulty with guitar playing, piano playing, typing, ball gripping, bicycle handlebar gripping, and drumstick grip. However, he continued school attendance and music activities. At the final follow-up, the QuickDASH (Quick Disabilities of the Arm, Shoulder and Hand) disability/symptom score was 4.5, and the music/performing arts module score was 0. Although these questionnaire scores were low, objective residual deformity, DIP stiffness, and difficulty with several specific activities persisted. Final radiographs confirmed maintained alignment and fracture union. The little finger DIP joint became ankylosed, whereas residual mobility remained in the ring finger DIP joint (Figure 7 and Figure 8). The final clinical and functional outcomes are summarized in Table 2.

## 3. Discussion

Mallet finger injuries are common; however, most are closed injuries that typically involve either a tendinous lesion or a single bony avulsion fragment at the dorsal base of the distal phalanx [3,4]. To our knowledge, although double-level and open mallet injuries have been reported, their combination in a single lesion has rarely been documented. This injury pattern is particularly unusual when intraoperative assessment demonstrates disruption not only of the tendon-attached fragment but also of the bony bed to which it should normally be reduced. In the present case, the ring finger showed an open double mallet lesion. Intraoperatively, the extensor tendon remained attached to a tiny avulsion fragment, whereas a separate dorsal base fragment was also present, indicating disruption of both the tendon-attached fragment and its bony bed. In addition, the little finger had a concomitant open fracture with substantial soft tissue damage. The clinical value of this case lies in showing how recognition of this combined tendon-side and bony-bed disruption, together with adjacent complex little finger injury, influenced both fragment identification and the reconstructive strategy. Compared with the broader mallet finger literature, in which many injuries can be managed successfully with splinting or standard operative techniques, the present case represented a more complex injury pattern requiring individualized reconstruction [13,14].

The key technical point in this case was the recognition of a tendon-attached tiny avulsion fragment of the ring finger and its relationship to the fractured dorsal base. Previous reports of double-level mallet injuries have emphasized that the lesion consists of both a larger dorsal fragment and a tiny tendon-attached fragment, and that this configuration may be difficult to diagnose preoperatively, particularly when the tiny fragment is not clearly visible [6,7]. In the present case, preoperative computed tomography was useful for identifying the osseous fragment configuration and raising suspicion of a double-level injury pattern. However, definitive understanding of the tendon attachment and the extent of disruption of the bony bed was achieved only through intraoperative inspection. Intraoperative inspection further clarified that the extensor tendon was attached to the smaller fragment and showed that this fragment was even smaller than anticipated. This distinction was clinically important because fixation of only the larger dorsal fragment would not necessarily restore extensor continuity. Moreover, the bony bed, to which the tendon-attached fragment should normally be reduced, was disrupted. This diagnostic challenge is consistent with the broader mallet fracture literature, in which fragment morphology and reducibility can substantially influence fixation strategy, particularly when the dorsal fragment is small, displaced, or difficult to control [15].

In this case, a more elaborate construct was required because both the tendon-attached fragment and its bony bed were disrupted. This configuration meant that fixation of the larger dorsal fragment alone would be insufficient to restore extensor continuity. Provisional stabilization was necessary, and extension-block pinning, transarticular pinning, and pull-out fixation were used to maintain reduction and restore extensor continuity. Thus, the combined construct was selected to address both osseous alignment and tendon-side stability in the setting of an unstable open injury with multiple small fragments. Reported alternatives for bony mallet injuries include extension-block pinning alone, hook plate fixation, tension band wiring, and open reduction techniques with direct tendon repair or suture-based fixation [13,15]. In the present case, however, fixation directed only at the larger dorsal fragment or tendon-side repair alone was considered unlikely to restore both extensor continuity and stable reduction because the tendon-attached fragment was extremely small and the bony bed itself was disrupted. Serial radiographs showed maintained alignment and progressive healing, suggesting that the combined construct provided sufficient initial stability. Recent reports of modified extension-block constructs for bony mallet fractures have likewise emphasized construct stability as a major goal, although improved stability does not necessarily translate into superior functional recovery [16]. In this respect, the present case differed from routine bony mallet fixation because the operative objective extended beyond joint reduction alone to restoration of tendon-side continuity in an open double-level injury [15,16].

The little finger required a different strategy because the distal fragment was too small for stable fixation that would avoid crossing the DIP joint. A non-transarticular fixation method was considered preferable; however, this was impractical because of the fragment size. Cross-pinning through the distal phalanx from the distal side was therefore performed. At the final follow-up, the DIP joint of the little finger was ankylosed; however, no major soft tissue complications, such as infection, skin breakdown, or nail deformity, were observed. Although functional limitations remained, the patient was able to continue school attendance and music-related activities. In this context, ankylosis of the little finger DIP joint may represent a trade-off for stability in this complex injury setting.

The final outcome for the ring finger was imperfect. Residual extension lag, limited DIP motion, coronal deformity, and sensory disturbances persisted. Nevertheless, some DIP mobility was preserved, and there was no infection, major skin complications, or nail deformity. Thus, the outcome may be considered functionally acceptable, but clearly suboptimal in terms of restoration of DIP motion and fine motor performance. These findings suggest that in such unusual open injuries, the primary surgical goal should not necessarily be the restoration of normal anatomy or full motion, but rather the preservation of a functional digit with acceptable alignment, soft tissue healing, and activity tolerance. In the present case, the patient was able to continue school- and percussion-related activities despite persistent limitations in fine motor tasks and gripping activities. The low QuickDASH disability/symptom score and music/performing arts module score should therefore be interpreted with caution. These patient-reported outcome measures may not fully capture persistent impairment related to isolated DIP joint deformity, stiffness, and task-specific limitations in fine motor control or forceful grip. His continued school attendance and music-related activities may have contributed to low perceived overall disability despite clear residual objective deficits. Accordingly, the patient-reported scores should be interpreted together with the clinical findings rather than as evidence of complete functional recovery. This interpretation is also supported by prior studies in which patient-reported hand function after mallet surgery was often favorable despite residual stiffness, extensor lag, or continued postoperative management constraints [13,17].

The mechanism underlying this unusual fragment configuration could not be determined with certainty in this case. Previous reports on double-level mallet injuries have suggested either a sequential injury process or separate injury events [6,7]. The coexistence of a very small tendon-attached fragment and a separate dorsal base fragment in the present case may therefore reflect a complex injury sequence rather than a simple single avulsion event.

This report has several limitations. As this was a single-case report, the reproducibility and generalizability of this strategy remain uncertain. In addition, because of the concomitant little finger injury, it was difficult to determine which component of the treatment contributed most to the final functional outcome. The coexistence of complex adjacent little finger trauma also confounded interpretation of patient-reported disability and hand function. Furthermore, postoperative rehabilitation was not conducted under a fully standardized single-institution protocol throughout the entire follow-up period.

Nevertheless, this case suggests that in selected open ring finger double mallet injury patterns, especially when the tendon-attached fragment is very small and the surrounding soft tissue injury is extensive, the possibility of bony bed disruption should be considered. In such situations, fixation of the avulsed fragment alone may be insufficient.

## 4. Conclusions

This case highlights that an open double mallet lesion may involve disruption of both the tendon-attached avulsion fragment and the bony bed. In such situations, careful imaging assessment, intraoperative fragment identification, and staged fixation may be important because fixation of the avulsed fragment alone may be insufficient. In selected injury patterns, provisional stabilization of the base may be useful before definitive tendon-side repair. Although residual deformity and limited DIP motion remained, the functional digit was preserved.

## Figures and Tables

**Table 1:** Clinical timeline of the present case.

Timepoint	Treatment/Event	Clinical Findings	Imaging/Outcome
Day 0	Emergency irrigation, debridement, and fixation	Open injuries of the ring and little fingers	Preoperative radiographs/CT obtained
POD 3	Occupational therapy started	Early postoperative pain controlled	-
6 weeks	All K-wires and pull-out suture removed	No major wound complication	Alignment maintained
3 months	Follow-up	Daily activities largely recovered; residual DIP stiffness remained	Progression of fracture healing
9 months	Follow-up	Residual functional impairment persisted	Continued union/remodeling
21 months	Final follow-up	Residual deformity and limited DIP motion remained; no infection or nail deformity	Final alignment/union confirmed

POD, postoperative day; CT, computed tomography; DIP, distal interphalangeal joint.

**Table 2:** Final outcomes at the last follow-up (21 months).

Parameter	Finding
Ring finger DIP motion	40° extension lag, 45° flexion
Little finger DIP motion	10° extension lag, 20° flexion
Grip strength	41.3 kg (unaffected side)/30.6 kg (affected side)
Sensory status	Residual dorsoulnar sensory dullness remained in both digits
Infection	None
Nail deformity	None
Skin complication	None
Functional limitations	Difficulty with guitar, piano, typing, ball grip, bicycle handlebar grip, and drumstick grip
Return to activity	Continued school attendance and music activities
QuickDASH disability/symptom score	4.5
QuickDASH music/performing arts module	0

DIP, distal interphalangeal joint; QuickDASH, Quick Disabilities of the Arm, Shoulder and Hand.

## Data Availability

The data presented in this study are available on request from the corresponding author due to ethical considerations.

## Abbreviations

The following abbreviations are used in this manuscript:

CT	Computed tomography
DASH	Disabilities of the Arm, Shoulder and Hand
DIP	Distal interphalangeal
K-wire	Kirschner wire
PIP	Proximal interphalangeal
QuickDASH	Quick Disabilities of the Arm, Shoulder and Hand
